# The SALT—Readout ASIC for Silicon Strip Sensors of Upstream Tracker in the Upgraded LHCb Experiment

**DOI:** 10.3390/s22010107

**Published:** 2021-12-24

**Authors:** Carlos Abellan Beteta, Dimitra Andreou, Marina Artuso, Andy Beiter, Steven Blusk, Roma Bugiel, Szymon Bugiel, Antonio Carbone, Ina Carli, Bo Chen, Nadim Conti, Federico De Benedetti, Shuchong Ding, Scott Ely, Miroslaw Firlej, Tomasz Fiutowski, Paolo Gandini, Danielle Germann, Nathan Grieser, Marek Idzik, Xiaojie Jiang, Wojciech Krupa, Yiming Li, Zhuoming Li, Xixin Liang, Shuaiyi Liu, Yu Lu, Lauren Mackey, Jakub Moron, Ray Mountain, Marco Petruzzo, Hang Pham, Burkhard Schmidt, Shuqi Sheng, Elisabetta Spadaro Norella, Krzysztof Swientek, Tomasz Szumlak, Mark Tobin, Jianchun Wang, Michael Wilkinson, Hangyi Wu, Feihao Zhang, Quan Zou

**Affiliations:** 1 Physik-Institut, University of Zurich, Winterthurerstrasse 190, 8057 Zurich, Switzerland; carlos.abellan.beteta@cern.ch; 2 Physics Department, Syracuse University, 900 South Crouse Ave, Syracuse, NY 13244, USA; dimitra.andreou@cern.ch (D.A.); martuso@syr.edu (M.A.); arbeiter@syr.edu (A.B.); sblusk@syr.edu (S.B.); sding09@syr.edu (S.D.); seely@syr.edu (S.E.); dgerma01@syr.edu (D.G.); zli59@syr.edu (Z.L.); xliang06@syr.edu (X.L.); lgmackey@syr.edu (L.M.); rjmounta@syr.edu (R.M.); hpham02@syr.edu (H.P.); miwilkin@syr.edu (M.W.); hwu107@syr.edu (H.W.); 3 Faculty of Physics and Applied Computer Science, AGH University of Science and Technology, al. Mickiewicza 30, 30-059 Krakow, Poland; roma.dasgupta@cern.ch (R.B.); szymon.bugiel@cern.ch (S.B.); firlej@agh.edu.pl (M.F.); tomasz.fiutowski@agh.edu.pl (T.F.); idzik@ftj.agh.edu.pl (M.I.); wokrupa@agh.edu.pl (W.K.); jmoron@agh.edu.pl (J.M.); szumlak@agh.edu.pl (T.S.); 4 INFN Sezione di Milano, Via Celoria 16, 20133 Milano, Italy; antonio.carbone@mi.infn.it (A.C.); nadim.conti@mi.infn.it (N.C.); federico.debenedetti@mi.infn.it (F.D.B.); paolo.gandini@cern.ch (P.G.); marco.petruzzo@mi.infn.it (M.P.); elisabetta.spadaro.norella@cern.ch (E.S.N.); 5 Institute of High Energy Physics, Chinese Academy of Sciences, 19B Yuquan Road, Shijingshan District, Beijing 100049, China; ina@cern.ch (I.C.); cbo@hnu.edu.cn (B.C.); nathan.allen.grieser@cern.ch (N.G.); jiangxj@ihep.ac.cn (X.J.); liyiming@ihep.ac.cn (Y.L.); liusy@ihep.ac.cn (S.L.); luy@ihep.ac.cn (Y.L.); shengshuqi@ihep.ac.cn (S.S.); marktobin1976@gmail.com (M.T.); jwang@ihep.ac.cn (J.W.); zfh@hnu.edu.cn (F.Z.); zouquan@ihep.ac.cn (Q.Z.); 6 Physics and Micro Electronic College, Hunan University, Lushan Road (S), Yuelu District, Changsha 410082, China; 7 European Organization for Nuclear Research, CERN, CH-1211 Geneve, Switzerland; burkhard.schmidt@cern.ch

**Keywords:** ASIC, front-end, ADC, DSP, PLL, DLL, SEE

## Abstract

SALT, a new dedicated readout Application Specific Integrated Circuit (ASIC) for the Upstream Tracker, a new silicon detector in the Large Hadron Collider beauty (LHCb) experiment, has been designed and developed. It is a 128-channel chip using an innovative architecture comprising a low-power analogue front-end with fast pulse shaping and a 40 MSps 6-bit Analog-to-Digital Converter (ADC) in each channel, followed by a Digital Signal Processing (DSP) block performing pedestal and Mean Common Mode (MCM) subtraction and zero suppression. The prototypes of SALT were fabricated and tested, confirming the full chip functionality and fulfilling the specifications. A signal-to-noise ratio of about 20 is achieved for a silicon sensor with a 12 pF input capacitance. In this paper, the SALT architecture and measurements of the chip performance are presented.

## 1. Introduction

The Large Hadron Collider (LHC) provides proton–proton collisions, at the centre of mass energies up to $\sqrt{s}=13$ TeV, which are studied by four large experiments: ATLAS [1], CMS [2], LHCb [3], and ALICE [4] (focused mainly on ion-ion collisions). New Physics phenomena may be studied via direct searches (production of new particles) or indirect ones where we concentrate on precise measurements of selected observables and compare the results with the Standard Model predictions. In both scenarios, the task of discovering New Physics requires, for instance, very precise and robust tracking systems that are able to provide a momentum measurement with very high resolution (typically better than one percent). This in turn, sets also stringent demands on the quality and functionality of the readout electronics.

The upgrade of the LHC machine will be capable of delivering more than an order of magnitude higher luminosity than presently utilised by the Large Hadron Collider beauty (LHCb) detector [5]. To conform with higher luminosity and the fully software-based trigger, all sub-detectors require front-end electronics that provide readout at the LHC bunch-crossing rate of 40 MHz [6].

The Upstream Tracker (UT) is a new sub-detector within the LHCb experiment. It plays a critical role in the software trigger, enabling a fast estimate of the momentum of charged particles produced in the pp collisions. It is based on silicon strip sensors organised in two pairs of planes with a small 5${}^{\circ }$ tilt between the strips within a pair [6]. The detector area is predominantly covered by 97 mm $p^{+}$–in–*n* strips with 190 μm pitch. In the vicinity of the beam pipe sensors doping is reversed to $n^{+}$–in–*p* for high radiation resistance and the density of the strips is doubled. Additionally, in the central area with the highest occupancy of particles, the strip length is reduced in half to reduce ambiguities in the pattern recognition.

All strip sensors in the UT will be read out by a new dedicated 128-channel Application Specific Integrated Circuit (ASIC) called SALT (Silicon ASIC for LHCb Tracking) [7]. The specifications of the SALT, based on the expected sensor characteristics and the operating conditions, are shown in Table 1.

The SALT chip is one of the first multi-channel low-power readout ASICs deployed in an High Energy Physics (HEP) experiment that features a fast Analog-to-Digital Converter (ADC) in each channel. Such an architecture was not possible to realise in the past, mainly due to high power consumption of the ADC. Recently, with advanced CMOS technologies and development of very low-power ADC architectures, it has become possible, and different groups started to develop readout ASICs [8,9,10,11,12] using such architecture. The latest trend is to add, beyond the amplitude measurement, timing information to the readout [8,11,12]. In addition to the challenges of low power, large number of channels, and radiation resistance, the SALT is the first readout ASIC in HEP experiments capable of digitising and processing data in each channel with the LHC bunch crossing frequency of 40 MHz. Moreover, it can cope with 100% hit occupancy in a single channel thanks to a dedicated shaping of the analogue signal with a very short pulse tail.

This paper is focused on the Silicon ASIC for LHCb Tracking (SALT) design and contains a basic set of test results, to verify the functionality of the chip. Section 2 describes the overall SALT architecture and the design of its main blocks. In Section 3, the test results of the chip are discussed.

## 2. Materials and Methods

### 2.1. SALT Architecture Overview

**Figure 1:**
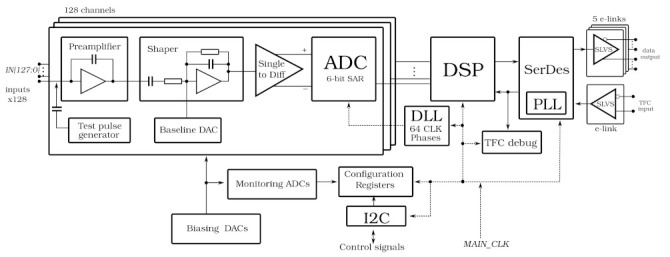
The SALT ASIC block diagram.

The block diagram of SALT is shown in Figure 1. The ASIC was designed in CMOS 130 nm technology offering good radiation resistance and low power consumption at sufficient speed. The SALT extracts, shapes and digitises analogue signals from the sensor, performs Digital Signal Processing (DSP), and transmits serially the output data. It uses an innovative architecture comprising an analogue front-end with fast and symmetrical shaping and a 40 MSps 6-bit ADC in each channel.

To synchronise the ADC sampling time to that of the pp collisions, a dedicated ultra-low power (0.7 mW @ 40 MHz) Delay-Locked Loop (DLL) circuit is used to shift and align an external clock. The digital ADC output is processed by the DSP block, which performs pedestal subtraction, Mean Common Mode (MCM) subtraction, and a Zero Suppression (ZS). Subsequently, the data packets are created and recorded in a local on-chip memory, which serves as a derandomising buffer. The data is then serialised via a number of data links called e-links [13]. The actual number of active e-links is between 3 and 5, and is set based upon utilising expected fraction of the output bandwidth while avoiding permanent memory overflow. In the UT detector, the number of active e-links is static for a particular chip and is established according to the expected data rate.

The SALT is controlled via the LHCb common protocol consisting of two interfaces: the Timing and Fast Control (TFC) and the Experiment Control System (ECS) [14]. The TFC interface delivers fast commands synchronised with the 40 MHz experiment clock (a byte including 0–8 commands every clock cycle), while the ECS serves to configure and monitor the ASIC. The former is realised through an input e-link and the latter by an Inter-Integrated Circuit (I^2^C) interface.

The SALT is a System-on-Chip (SoC) type ASIC. In addition to the previously mentioned blocks and functionalities, it encompasses: reference voltage generators in each analogue front-end channel, several 5–8 bit Digital-to-Analog Converter (DACs) to set biasing in different analogue blocks, several 6-bit monitoring ADCs for selected circuitry such as DACs, DLL or PLL. The last functionality is also important for monitoring the ASIC performance over time, which is especially important in the high radiation environment. Moreover, various techniques were used to make the ASIC resistant against radiation induced effects like TID and Single Event Effect (SEEs) [15].

The design presented below reflects the third SALT prototype. Previous prototypes exhibited a large 40 MHz disturbance at the output when the front-end was connected to an input capacitance of several picofarads, and the disturbance increased with the input capacitance value. The deterioration occurred after the transition from an 8-channel to a 128-channel chip, since no such disturbance was observed for the 8-channel ASIC prototype [16]. A number of modifications regarding the power distribution, analogue blocks and layout were been made to eliminate this effect, and are not discussed in detail here.

### 2.2. Analogue Front-End

**Figure 2:**
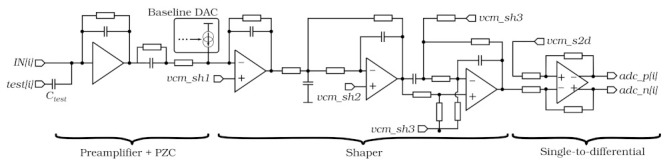
Simplified block diagram of analogue front–end.

The analogue front-end block diagram is shown in Figure 2. It comprises a charge-sensitive preamplifier with pole-zero cancellation, a three-stage shaper, and a single-ended to differential converter. A fast peaking time of the shaper $T_{peak}\approx 25$ ns is required. Moreover, the signal must return to baseline quickly to minimise the pile-up and spill-over in the next bunch crossing, thus allowing clear separation between subsequent pulses at the LHC bunch crossing rate of 40 MHz. In addition, a very low power consumption 1–2 mW/channel is required. The front-end must function well with a sensor capacitance between 1.6–12 pF and input signals of both polarities, providing a signal-to-noise ratio above ten (S/N >10). The last block in the analogue chain converts a single-ended shaper output to a differential signal which is then sent to the ADC.

One of the biggest challenges for this block was to obtain a very short tail of the shaper output pulse (about 5% amplitude after $2T_{peak}$) with minimal power consumption. Preliminary studies showed that this goal is not achievable with a standard semi-Gaussian shaping using a reasonable number of real poles in the transfer function. Therefore, a more advanced three-stage shaping, based on complex poles and zeros in the transfer function [17] was implemented. The first stage has a real pole; the second, in the multiple negative feedback configuration, has two complex poles, while the third one, in Boctor configuration [18], has two complex poles and two complex zeros. The shaper transfer function was optimised in simulations to get the most symmetrical shape of the output pulse.

All shaper stages are referred to the same common voltage value (*vcm_sh*), although generated separately for each of them (*vcm_sh1*, *vcm_sh2*, *vcm_sh3*). The same common voltage value is also generated in the single-ended to differential converter (*vcm_s2d*). A common 6-bit DAC is used for the common voltage setting. Moreover, the bias currents of all front-end blocks (preamplifier, shaper, single-to-differential converter) can be adjusted using a dedicated internal 5-bit DACs. Additionally, every channel contains an 8-bit trim DAC for precise baseline setting. To mimic the pulses from a silicon sensor, each channel is equipped with a small (100 fF) test capacitance connected to an internal pulse generator. The amplitude of the pulse is controllable via another dedicated 6-bit DAC. This is an important feature in the testing the response of the SALT over the full dynamic range.

### 2.3. Analog-to-Digital Converter (ADC)

**Figure 3:**
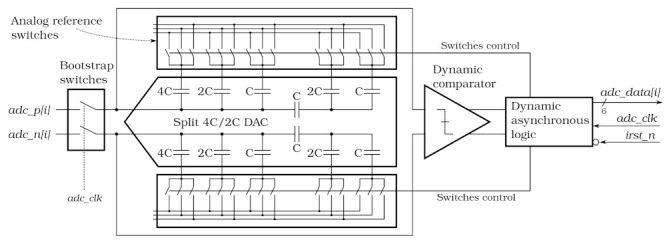
Block diagram of the 6-bit Successive Approximation Register (SAR) Analog-to-Digital Converter (ADC).

A fully differential Successive Approximation Register (SAR) ADC converts the analogue signal to the digital domain. The ADC provides 6-bit resolution with a 40 MSps sampling rate. One of the most important constraints of the ADC design is to keep the power consumption significantly below 1 mW, which drove the design to use a SAR ADC architecture. To further reduce the dissipated power, a Merged Capacitor Switching (MCS) scheme [19] was implemented. Since in the given technology the best capacitance matching is offered by a metal-insulator-metal capacitors, which are relatively large, a split capacitor DAC approach was used, as shown in Figure 3.

In addition to the DAC switching scheme, two other features are implemented to reduce the power consumption: a dynamic comparator [20] and an asynchronous control logic, as shown in Figure 3. The dynamic comparator dissipates power only during the bit cycling process, while the main power saving in the asynchronous control logic comes from the fact that the fast clock signal for the conversion of subsequent bits is eliminated. Finally, additional power reduction is achieved by implementing part of the logic with dynamic flip-flops. At the input, bootstrapped switches [21] are used; although they costs some extra power, they increase the linearity of the ADC. More details on the ADC design and its multi-channel performance can be found in references [22,23].

To improve robustness against SEE events, the ADC control logic in the SALT was slightly modified. The sampling signal launches the ADC reset sequence regardless of whether the previous conversion was finished or stopped due to a SEE. Even though it causes the loss of two ADC samples for SEE event (one caused directly by SEE and the next one when the reset sequence is performed) it ensures continuous operation.

Due to the fully asynchronous architecture of the ADC, the current consumption between conversions is almost zero. It is roughly uniform during the conversion which takes slightly more than half of the 40 MHz clock period. As a result, the current drawn by the ADC is approximately a square wave with roughly 50% duty cycle and amplitude twice as large as the average consumption. Such a pattern, amplified by all 128 ADC channels working synchronously can be an irremovable source of disturbances induced in the analogue front-end. One possible solution to this problem could be an artificial delay after each bit cycle increasing effectively the overall conversion length. However, the time structure of the current consumption in such a case would consist of large spikes, during each bit cycle, and the gaps in between. Moreover, the increased conversion length may cause issues at higher irradiation doses due to expected slowdown in ADC control circuitry. Therefore a definitely less sophisticated, yet robust approach was chosen. Since the averaged ADC power consumption is far below the ASIC specification, a circuitry drawing additional dummy current between the conversions was added to each ADC. This increases the average current, but makes it uniform in time. The additional current can be disabled by the ECS command, reducing the total power consumption, but increasing the probability of the interference with the analogue part of the ASIC.

### 2.4. Delay-Locked Loop (DLL)

The 40 MHz main clock delivered to SALT as a part of the TFC protocol is synchronous with the beam. However, its phase, although constant, is difficult to predict in advance. Therefore, it is shifted in the dedicated low power DLL to obtain a precise alignment between the ADC sampling phase and the bunch crossing. The DLL produces a second replica of the 40 MHz clock, which is used to tune the delay of the internal test pulse for the analogue front-end.

**Figure 4:**
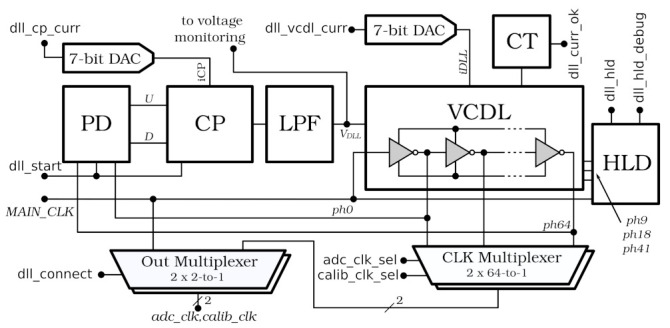
Delay-Locked Loop (DLL) block diagram: PD—Phase Detector, CP—Charge Pump, LPF—Low Pass Filter, VCDL—Voltage Controlled Delay Line, CT—Current Test, HLD—Harmonic Lock Detector.

Figure 4 shows the block diagram of a DLL. The central block is the Voltage Controlled Delay Line (VCDL), which includes 64 delay cells and creates 64 independent clock phases. Two of them (for the ADCs and the internal test pulse generators) are selected by a pair of independent multiplexers (CLK Multiplexer) connected directly to the VCDL. These signals are delivered to the second pair of multiplexers (Out Multiplexer) which select between them or main clock (*MAIN_CLK*). This bypass function is utilised during the DLL synchronisation process to avoid randomly changing phases in the outputs. More details on the DLL core design can be found in [24].

A DLL is relatively sensitive to variations of Process, Voltage, and Temperature (PVT) parameters. Although the feedback loop can compensate for small changes of temperature and voltage, to cover a full range of PVT variations, biasing currents have to be adjusted. In the SALT, two 7-bit voltage DACs controlling biasing currents of the charge pump and the VCDL were implemented for this purpose. The VCDL is significantly more sensitive to PVT changes and its biasing current has to be adjusted for each chip. The Current Test (CT) logic was added to simplify this procedure. This block is active only during the VCDL configuration procedure and allows for the determination of the optimal value of the VCDL current. It measures the time delay of *ph32* (one of the 64 VCDL outputs, placed exactly in the middle of the delay line) using *MAIN_CLK* as the sampling clock. When the time delay of *ph32* is around half of the *MAIN_CLK* period, the CT logic sets the high state to output *dll_cur_ok*.

In the case of the significant change of some PVT parameters the VCDL may be out of range or DLL may lock to “harmonic” delay, that is, a delay which is a multiple (2, 3, or more) of the 25 ns reference period. The first case is easy to detect with the control voltage *V${}_{DLL}$* monitoring (see Section 2.8), but the second one is more demanding. To address this issue, a Harmonic Lock Detector (HLD) circuit was designed and implemented.

The HLD circuit checks the phase relation between main clock and VCDL outputs: *ph9*, *ph18*, and *ph41*. On the contrary to the CT logic, in the HLD block *MAIN_CLK* is sampled at the rising edge of the aforementioned signals. The result is available as a 3-bit signal *dll_hld_debug* and may be read from one of the SALT registers. When the DLL is synchronised properly, *dll_hld_debug* is equal to 110. An additional single bit *dll_hld*, informs the user about the harmonic lock status.

### 2.5. Digital Signal Processing (DSP)

The digital part of the SALT is clocked directly by the 40 MHz experimental clock, so its phase is different from the ADC sampling clock phase. Hence, before any data processing, the samples from ADC have to be handed over to the DSP clock domain. The common solution in such a situation, an asynchronous FIFO queue, is not a good choice for the SALT ASIC. First, although the relation between ADC and digital clocks may vary, they are not asynchronous since the phase shift is known (defined in the chip configuration). Second, in the radiation environment, some ADCs may incidentally receive an additional sampling pulse (because of SEE) permanently destroying data coherence (some FIFOs would be shifted). Therefore, a simple circuit consisting of two flip-flops connected in parallel, sampling data on a positive and negative clock edge, respectively, is used. At least one of the flip-flops samples ADC data correctly for any phase relation. For most cases both work correctly but the optimal one should be chosen taking into account timing margins. This solution makes the data coherence immune to SEEs.

**Figure 5:**
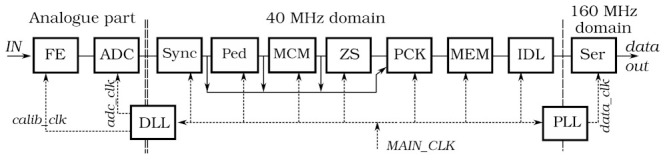
Data processing in the SALT; vertical lines separate clock domains; FE—analogue front-end, ADC—Analog-to-Digital Converter, Ped—Pedestal Subtraction, MCM—Mean Common Mode, ZS—Zero Suppression, PCK—Packet Builder, MEM—data memory, IDL—Idle Packets Generator, Ser—Serialiser, PLL—Phase-Locked Loop, DLL—Delay-Locked Loop.

The ADC samples are encoded as 6-bit two’s complement numbers. Signed numbers are necessary to cover both sensor polarities. The negative values are obtained for $p^{+}$–in–*n* sensors and merely for such samples an arithmetic inversion is performed. Thereby, the rest of the DSP chain, shown in the central part of Figure 5, was designed based on the assumption that all samples are positive.

The first DSP operation is the masking of a noisy or dead channel. Such channels are completely removed from further data processing. In the next processing step, the pedestals, uploaded previously to the configuration registers, are subtracted. Therefore, in channels without a signal from the sensor (without a hit), the expected pedestal-subtracted value is zero. A common-mode disturbance could shift all channels coherently up or down. To remove this common-mode shift, a mean common mode MCM offset is computed, and subtracted. Only those channels with amplitude below a pre-defined MCM threshold contribute to the average. The choice of MCM threshold is a trade-off between the largest rejected disturbance and the smallest signal which may be detected. Both subtractions in pedestals and MCM are fragile operations since they may easily overflow their 6-bit representation. Hence, the saturation arithmetic is utilised here, i.e., the result is limited to the range between a minimum and maximum value of 6-bit two’s complement number.

The following step is the ZS, which eliminates all signals smaller than or equal to a non-negative ZS threshold (roughly 3–4 times the expected noise). At this stage, the ADC values are rounded to 5-bit precision because the negative part is no longer needed. According to simulations, the occupancy is about 1% or less, and therefore the ZS provides a very large reduction in the data that needs to be processed and transmitted.

Data from the ZS block are sent directly to the packet builder (PCK), which creates data packets with the format specified in Table 2. The packets are stored in design-specific memory (MEM) in advance of their serialisation. During normal operation, the ASIC generates mostly packets with data compressed in the ZS block (*Normal* packets). However, for diagnostic and testing purposes, it is important that the chip can also transmit packets without compression, called *NZS* (Non-Zero Suppressed) packets, in response to a fast TFC command. The NZS packet contains raw ADC values or partially processed data (see alternative input of PCK block in Figure 5). Moreover, it includes the values calculated by the MCM algorithm, i.e. the number of channels without a hit and the MCM average value. Both parameters are very useful for DSP testing and debugging.

The SALT generates exactly one data packet every clock cycle. To enable tracking and loss detection in the DAQ system, each packet contains four least significant bits of Bunch Crossing Identification (BXID) counter in the first field of its header. The sole exception is the synchronisation packet (*Sync*) which contains the full 12-bit BXID value. This high priority packet, similarly to other special packets like *HeaderOnly* and *BxVeto*, causes the data to be dropped. The packet type depends mainly on the current TFC command, but the number of hits and free memory space (the next element in the data processing chain) are also taken into account. When a *Normal* packet is expected but the number of hits (*nHits*) is larger than 63, a *BusyEvent* is created instead. The packet builder checks whether *Normal* or *NZS* packets will fit into memory, and if not, a *BufferFull* or *BufferFullN* status is created, respectively.

The on-chip memory (MEM) collects built data packets. Their size may vary in consecutive clock cycles, from one (e.g., *HeaderOnly*) to 67 (*NZS*) 12-bit words. Therefore, the memory works as a derandomising buffer. In the same clock cycle, the serialiser receives a constant amount of data, which size depends on the number of active data links. More details of the memory design, including the feedback to the packet builder, can be found in [25].

### 2.6. Serialiser and Deserialiser (SerDes)

The SALT receives TFC commands and transmits data packets to the DAQ at 320 Mbps via a number of differential e-links, based on the Scalable Low-Voltage Signaling (SLVS) interface [26]. The rate is obtained by fourfold multiplication of the main clock, performed by a dedicated PLL and DDR transmission. It is required that all active output e-links are continuously transmitting data packets [14]. Therefore, when the memory is empty, the chip transmits dedicated *Idle* packets (constant 12-bit word 0000_11_0000) to fill out the serialiser bandwidth.

**Figure 6:**
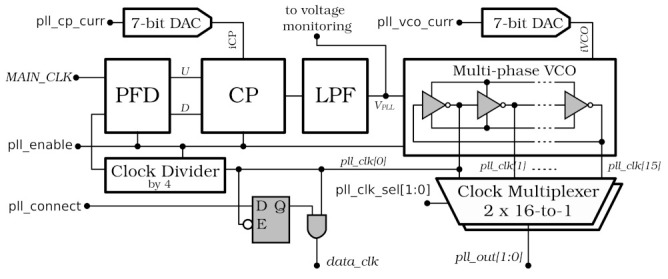
PLL block diagram: PFD—Phase and Frequency Detector, CP—Charge Pump, LPF—Low Pass Filter, VCO—Voltage Controlled Oscillator.

Since the synchronisation of the data stream is performed at the receiver side, the serialiser is relatively simple and a data synchroniser is implemented only at the deserialiser input. Because of DDR, there are two independent synchronisation circuits, one for odd and another for even bits. In each one, data are captured with a single input flip-flop connected to a clock with an adjustable phase generated by the PLL. A block diagram of the PLL is shown in Figure 6. Its basic architecture is rather typical [27]; however, it is highly customised and achieves an ultra-low power of 0.95 mW @ 160 MHz.

The Voltage Controlled Oscillator (VCO) generates 16 clock phases in which the first one, phase 0 (*pll_clk[0]*), clocks most of the SerDes circuitry. The other two, selected independently by two internal multiplexers (similarly as in the DLL), are connected merely to the two aforementioned input flip-flops in the deserialiser. Thereby, the deserialiser is quite flexible and can handle data streams with time shifts between even and odd bits.

The PLL operating conditions may be monitored by observation of VCO control voltage *V${}_{PLL}$*. This voltage can be tuned by a 7-bit DAC, which controls the VCO centre frequency, allowing for compensation in PVT variations.

### 2.7. Single Event Effect (SEE) Mitigation

Two kinds of radiation phenomenons are typically considered in ASIC design: cumulative (TID) and temporary (SEE) effects. The former affects mainly analogue circuitry changing transistor parameters, while the latter mostly affects digital blocks generating parasitic pulses. If the radiation induced pulse is created and propagated in combinational logic it is called Single Event Transient (SET) [28], but when it changes a memory element state, for example, a flip-flop (directly or indirectly by latched SET), it becomes a Single Event Upset (SEU) [29].

The 130 nm CMOS processes are considered as radiation resistant for moderate radiation doses. Therefore, no special provisions were applied against TID effects in the SALT analogue part, except for the 8-bit baseline trim DACs, drawing very small currents (<1 μA), where NMOS enclosed layout transistors [30] are used.

**Figure 7:**
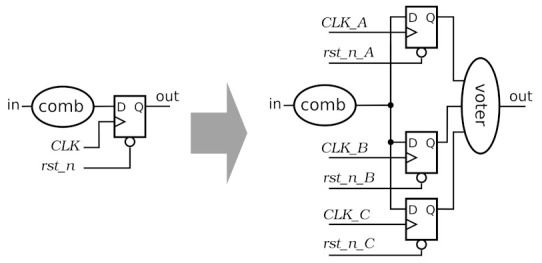
Triple Modular Redundancy (TMR) procedure applied in the SALT.

There are many different SEE mitigation techniques [31]. Most of them utilise some kind of redundancy to be able to recover the correct information in a radiation affected node or bit. For the digital circuitry in SALT, a partial Triple Modular Redundancy (TMR) [32] was chosen as more complicated approaches are impractical or impossible to implement due to the available power budget and limitations on the amount of material in the active detector volume. The mitigation of the effects of SEE was restricted to flip-flops and two critical global nets: clock and asynchronous reset, as shown in Figure 7. The TMR mitigates SEUs, which was recognised as the predominant source of radiation errors taking into account the technology and the main clock frequency [33,34]. Protection of global nets against SEU is crucial. Without it, a single SET in a clock or reset net can affect many flip-flops in the digital part.

The selected TMR architecture has some consequences. Functional blocks, such as the reset synchroniser or PLL, located inside the reset or clock trees, have to be triplicated with great care. To ensure the correct operation of SerDes block, each PLL output can be used as a fast clock only after its internal synchronisation (see *pll_connect* signal in Figure 6). Moreover, clock frequency and phase manipulation divide the chip in several clock domains, using triplicated synchronisers in several places (for details see [35]).

Configuration registers, in addition to the normal triplication, have also protection against SEUs, as they must keep their values for a long time. Each register has an error detection circuit, which refreshes it automatically when an error is detected. Registers are organised in blocks and each block is connected to the SEU counter, which is incremented by one when an error is corrected. The counters allow for the calculation of the SEU cross-section in real experimental conditions.

### 2.8. Internal On-Line Monitoring

On-line monitoring becomes a vital functionality for systems expected to operate for a long time in radiation environments, where the electronics is ageing additionally by TID accumulation. After several years or months (for higher doses) of operation, some ASIC parameters, in particular biasing currents, will have to be adjusted.

The SALT has several monitoring functions and blocks beyond the already described *NZS* data packets and SEU counters. The most important, for the reasons just discussed, is the 6-bit monitoring ADCs (same as in front-end channels) connected to almost all internal biasing DACs. These DACs set biasing of analogue front-end blocks (preamplifier bias current, shaper bias current, input test pulse amplitude, reference current for common mode voltages, single-to-differential converter bias current) and other functional blocks such as the SLVS biasing current and reference voltage. The user can check the actual current or voltage by reading the ADC output value via the I^2^C interface. The exceptions are the DACs located in the DLL and the PLLs, which are not directly monitored. However, their operation is checked indirectly by observation of the internal control voltages *V${}_{DLL}$* and *V${}_{PLL}$* in DLL and all PLLs, respectively (see Figure 4 and Figure 6).

The LHCb DAQ system specification [36] imposes additional monitoring requirements on the SALT. Each TFC command has a counter (32 or 48-bit long) and a snapshot register bounded to it. Counters keep running values; to read them, an additional TFC command called Snapshot was introduced. This command rewrites the current value of all TFC counters to snapshot registers from which the values may be read via the I^2^C protocol. The same mechanism was used for some other running parameters like free space in the memory buffer or number of sent *Idle* packets.

### 2.9. Layout and Integration

**Figure 8:**
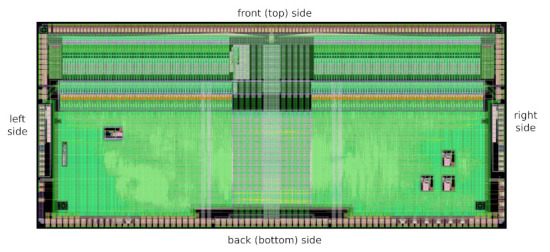
Layout of SALT ASIC; the die size is 10.905 mm × 4.75 mm.

The layout of the SALT is presented in Figure 8. It has dimensions of 10.90 mm× 4.75 mm, where the horizontal dimension is driven by the pitch of the input pads (80 μm) matching the UT silicon sensor pad pitch. Inside the chip, signals are processed from the input pads (top in Figure 8) towards the serialiser and data drivers, placed near the back side (bottom in Figure 8). The top part of the layout contains 128 readout channels, oriented vertically, with analogue front-end and ADC, while the bottom section is almost purely digital, including the PLLs (3 rectangles at bottom-right) and DLL (rectangle at centre-left) circuits. The central vertical area is left nearly empty to accommodate the power rails for the analogue blocks.

**Figure 9:**
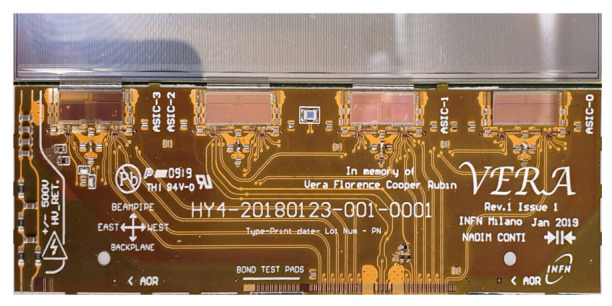
SALT mounted on the hybrid and connected to the sensor.

One of the most important issues concerning a multi-channel ASIC is the uniformity of analogue parameters in the front-end and ADC blocks. Apart from an unavoidable statistical spread arising from the ASIC production process, a systematic spread may also occur. Its main source originates from the voltage drop across the channels, due to the resistivity of the power rails. To minimise this spread, the power supply is typically delivered symmetrically from two sides of the ASIC. Unfortunately in case of SALT, due to space limitation on the printed circuit board (hybrid) hosting the ASICs, a different approach is taken. In the UT detector two types of hybrids are required, one containing four SALTs (shown in Figure 9) and another with eight SALTs chips, called 4-chip and 8-chip hybrids, respectively. Both hybrids have the same width, which has to (approximately) match the width of the sensor. The 8-chip hybrid is required to match the larger channel density in the high occupancy regions of the detector. Due to the dimensional constraints on the hybrid, there is insufficient space to make wirebonds between the ASICs and the hybrid in the region between the ASICs. Since it was decided to use one ASIC design for both hybrid types, the SALT layout must support two power supply schemes. For 4-chip hybrid the power supply is delivered symmetrically from the left and right sides (see Figure 8), while for 8-chip hybrid only from the back side. To keep a similar level of symmetry in both cases, the analogue part of the chip is divided into two 64-channel blocks, and the power supply is delivered centrally from the back side through the digital part of the chip and distributed in a star-like connection between the blocks.

The power supply scheme proposed for 8-chip hybrid can be very risky in a multichannel ASIC with an ADC in each channel, due to long supply rails which can not be well simulated. This is because standard design tools provide only the extraction of parasitic resistance and capacitance, but not inductance. However, the mutual inductance between long adjacent rails belonging to different power domains, that is, analogue, ADC and digital, becomes critical—and in extreme cases—may lead to instability of the entire system. To eliminate the inductive coupling between the analogue front-end and ADC blocks, the power supply of the latter was transferred to the digital domain (where a two-layer supply mesh with interleave is used) leaving the central part of the layout only for front-end power rails. Moreover, to reduce the sensitivity to pick up disturbances by the ground network of input transistors, dedicated insulating layers and guard rings were added [37].

## 3. Results and Discussion

The ASIC was manufactured, glued, and bonded to a prototype 4-chip hybrid, as shown in Figure 9. Although the figure shows the SALT already bonded to the silicon sensor, the first measurements were performed without it. Similar results were obtained for several chips, so the paper presents only the results for a representative one. Although these results verify the performance of the chip, they are not sufficient to qualify a complete detector system. Detailed measurements of the complete detector setup quantifying chip parameters on different hybrids will be presented in future publications.

**Figure 10:**
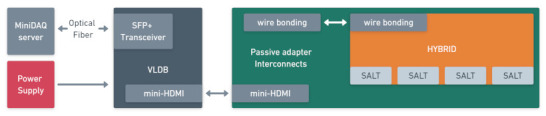
Block diagram of the DAQ used for SALT validation.

The test setup, as shown in Figure 10, partially mimics the expected readout in the LHCb experiment by using the final hybrid together with adapter boards acting as substitutes for the final detector parts. The hybrid, made in Flexible Printed Circuit Board (FPCB) technology with a ceramic stiffener, is connected to a Versatile Link Demo Board (VLDB) [38], through a passive interconnect adapter, which supports the hybrid mechanically and accounts for connectors and pinout differences. The VLDB delivers clock, TFC commands, I^2^C, receives data via e-links, and communicates through optical links with the next component in the test chain, the MiniDAQ [39]. The VLDB encompasses the elements chosen to be a common communication platform for the new generation of readouts for several LHC experiments. It was developed in cooperation of many groups focused around LHC to improve and standardise the testing of readout ASICs. On the contrary, the MiniDAQ is specific merely to LHCb experiment. It emulates the planned DAQ system on a small scale, integrating all of the key components packed inside a single Field-Programmable Gate Array (FPGA) [39]. The VLDB, and consequently the hybrid, are then connected to an Agilent Technologies N6705B DC Power Analyzer to monitor the current draw of the SALT and its correct behaviour depending on the registers’ configuration. The high speed SLVS signals, together with the I^2^C, are monitored at multiple points along the readout via an SDA808Zi-A 8 GHz Serial Data Analyzer to ensure the required signal integrity and bit-error rate (BER) are achieved. The overall setup is then positioned in a humidity, temperature, and particulate controlled clean room to guarantee consistency between different measurement sessions. All the tests described in this article were conducted in a standard temperature environment and then validated at the lower temperatures at which SALT is expected to operate.

### 3.1. Digital Tests and Chip Configuration

The testing procedure starts from checking the I^2^C communication and internal registers, which are necessary to perform all other steps. Write and read operation is performed for all registers several times with arbitrary patterns (e.g., 0x55 and 0xAA to inspect all the bits in the register). Prior to testing more advanced functions, the communication between VLDB and chip SerDes has to be established. First, all three PLLs are turned on and their control voltages are checked (via on-line monitoring) to confirm the correct lock. Next, PLLs are connected to SerDes and the links from chip to VLDB are configured. Their configuration is based on known patterns generated by the chip (uploaded via I^2^C). When communication from SALT to VLDB is stable, the ASIC is switched to a loop-back mode, where the data received by the deserialiser are directly sent back, and the deserialiser is configured. Finally, the chip is ready to receive TFC commands and send data packets. Hereafter, the data processing can be studied.

The digital processing is verified in a special test mode, where each DSP channel input sees the same constant value defined via the configuration register. Utilising *NZS* data packages to collect data directly from the output of the pedestal block is enough to check pedestal subtraction (as the channels are independent). The test confirms the proper operation of this block.

In the next step (also in the test mode), pedestals controlled individually for each channel are added to the inputs to create arbitrary patterns for the next MCM block. The *NZS* package then collects data from the output of the MCM block. Since the *NZS* package includes both the average MCM value and the number of channels, the correctness of MCM calculation is thoroughly verified. The tests performed for all possible average values and many different input sets confirmed the proper operation of this block.

The verification of the ZS block, the last DSP processing step is performed similarly to the previous test. Static inputs are generated based on the uploaded pedestal. The full range of *Normal* packet sizes was observed during the test, starting from packets without hits, up to the maximum number of 63 hits. For a larger number of hits, the chip creates *BusyEvent* packets as expected (see Table 2) confirming the correct operation of this part.

All digital processing tests described above were conducted for several thousand data packets. Therefore, to operate continuously while avoiding memory overflow, a specific stream of TFC commands needs to be utilised. As a result, the *Normal* or *NZS* data packets (later containing a large amount of data) are interleaved with a number of *HeaderOnly* packets. Decreasing the number of *HeaderOnly* packets, memory overflow can be checked. In this case, tests verified the data consistency and packet builder packet conversion from *Normal* or *NZS* to *BufferFull* or *BufferFullN*, respectively.

### 3.2. Measurements without Sensor

**Figure 11:**
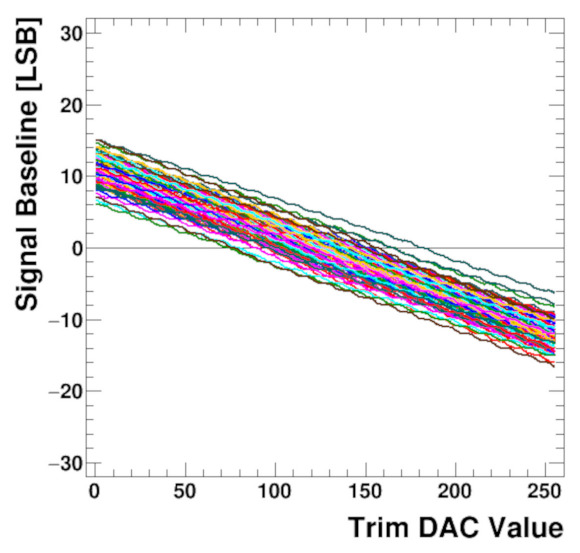
Baseline trim Digital-to-Analog Converter (DACs) scan for 128 channels before sensor connection.

To verify the analogue shaper together with the ADC and further digital processing, the digital output of all channels was measured as a function of the baseline trim DACs, as shown in Figure 11. To remove noise each point is calculated as an average of several thousand values. It is seen that the trim DACs work correctly in the whole range and their trim range is wide enough to correct the baseline spread. Although the trim DAC signal is static, this plot proves that the whole readout chain, starting from the shaper and ending at the serialiser, works correctly. A deeper look at the Figure 11 reveals small steps in the trim curves. This effect is caused by the higher resolution of the trim DACs over the ADCs.

**Figure 12:**
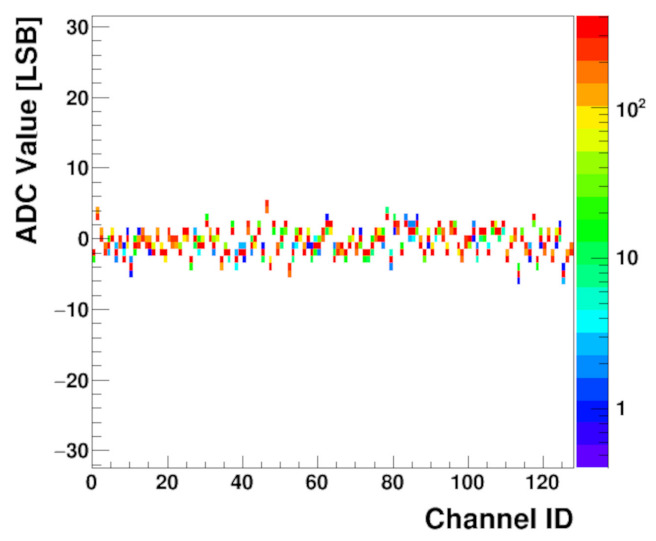
Baseline noise histogram for all trim DACs set to the default value 128; colour indicates bin height; sensor not connected.

The noise distribution for all channels is shown in the histogram in Figure 12, which was created from the same amount of data, but for a single trim DACs value of 128. For each channel, the ADC output value is nearly constant and the noise Root Mean Square (RMS) is significantly below one Least Significant Bit (LSB). Figure 12 also shows that the baseline spread is purely random.

**Figure 13:**
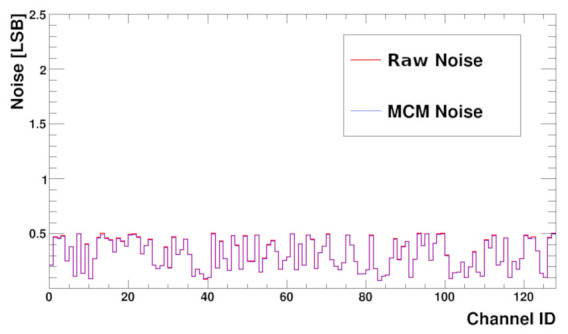
Baseline noise Root Mean Square (RMS) versus channel number before (Raw Noise) and after (MCM Noise) MCM correction; sensor not connected.

The noise before and after common mode subtraction is presented in Figure 13. The values are calculated from the raw data used to build the histogram described above. Raw noise is calculated from the RMS of the values. To obtain the MCM noise the pedestals and then the MCM are subtracted before RMS computation.

From the plot, it is seen that the measured noise RMS varies channel to channel from very low values up to half of LSB. For a uniform multi-channel ASIC one expects almost the same noise for each channel. However, this is not the real analogue noise but the result after digitisation. If the real noise is much smaller than the LSB of the ADC, then the RMS result after digitisation strongly depends on the position of the analogue channel baseline versus the closest ADC levels. If the baseline is exactly in the middle between two ADC levels the RMS should be half of LSB. On the contrary, if the baseline sits on ADC level, then the noise should be close to zero. Such behaviour is actually seen in Figure 13. In the SALT, it is possible to obtain uniform noise distribution from channel to channel by setting the baselines of all channels to the same level, applying the trim DAC correction (and assuming that the trim DAC precision is much better than the ADC one). This was also verified in measurements.

**Figure 14:**
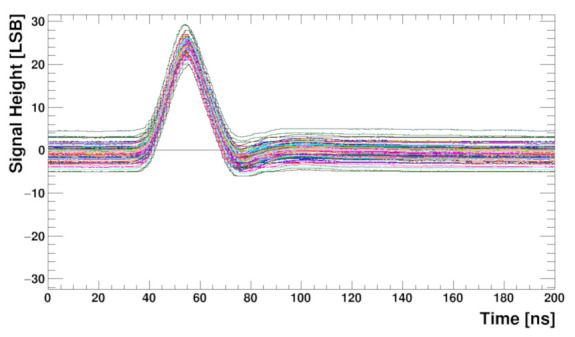
Pulse shapes for about 20 ke input charge and 128 channels obtained from the ADCs outputs. The sensor is not connected for this measurement.

To check the whole analogue front-end (including preamplifier) behaviour, measurements with the input pulse, generated internally by the calibration circuitry, were performed. Although in the presented architecture direct observation of the pulse shape is impossible, it may be reconstructed from a series of measurements done with different ADC sampling phases. The result is shown in Figure 14. The internal DLL is used to shift the sampling phase within a clock period (25 ns) in 0.4 ns steps while the digital delay is used to extend the range to several clock cycles. The plot shows responses of all channels to the injected charge equivalent to about 1 Minimum Ionising Particle (MIP) in a 250 μm thick silicon sensor. With such measurements the ADC sampling phase can be aligned to obtain a maximum pulse amplitude. Good symmetry of the pulse shape with a short tail, fulfilling the specification (about 5% of amplitude after twice the peaking time), may be observed.

After alignment of the ADC sampling phase, measurements were performed for the whole range of the internal input charge generator. The circuit was found linear for both input signal polarities between about −40 ke and 40 ke of input charge. The measurements show that the SALT ASIC works very well without the sensor connected to its inputs.

### 3.3. Measurements with Sensor

The third optimised SALT prototype with a biased UT sensor connected was characterised with the same set of measurements as discussed previously. A large sensor capacitance presents the most difficult conditions for the operation of the ASIC, and therefore, only results from the largest 12 pF/strip UT sensor (Type A) [40] are presented here (other sensor types were also tested).

**Figure 15:**
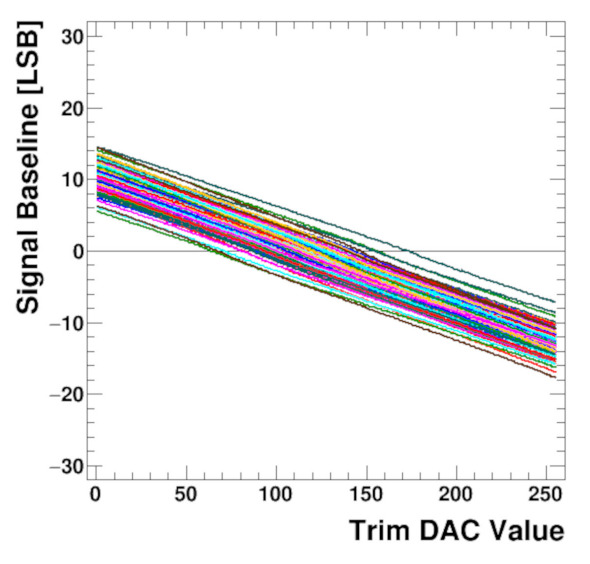
Baseline trim DACs scan for 128 channels after a biased UT sensor is connected.

**Figure 16:**
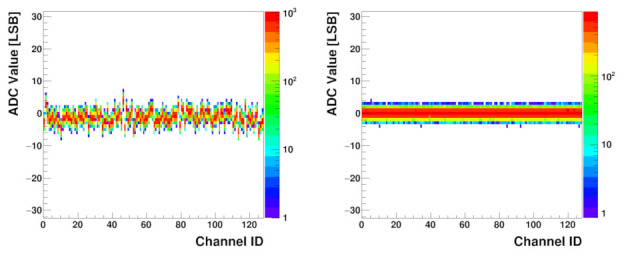
Baseline noise histogram before applying the trim DAC correction (**left**) and after the trim DAC correction (**right**), both with a biased UT sensor attached.

The curves obtained from the trim DACs scan are presented in Figure 15. They are more smooth in comparison to the results without sensor due to the larger noise, which is also seen in the baseline noise histograms shown in the Figure 16.

The left plot in Figure 16 shows the baseline noise histogram obtained for the default values of the trim DACs corresponding to the one in Figure 12. In both figures, the spread looks purely random between channels. The plot to the right shows the same noise after the trim procedure. It is clearly seen that the noise is very homogeneous between channels.

**Figure 17:**
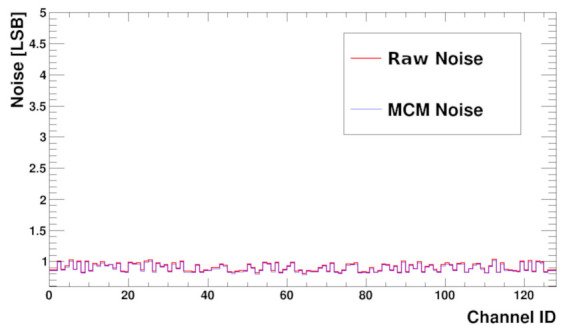
Baseline noise RMS versus channel number shown without and with MCM correction with a biased UT sensor connected.

The distribution of noise as a function of channel number (with a sensor attached) is presented in the Figure 17, before and after MCM subtraction. As expected, the noise RMS is higher than for the bare chip and is between 0.8–1 LSB. This is due to sensor capacitance. Presented results show the same values of noise before and after MCM subtraction, which not always must be the case. Measurement done with different setups gave the same MCM noise value, while the noise measured without MCM correction could be about 30% higher.

**Figure 18:**
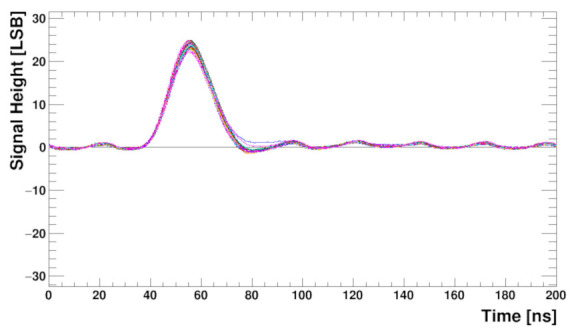
Pulse shapes for 128 channels obtained from the ADCs outputs after trim procedure applied, with a biased UT sensor connected.

The response of the front-end to the input signal is studied, similarly as for the setup without sensor, using the internally generated pulse and reconstructing the output pulse shape from the output data obtained with different ADC sampling phases. The results for all SALT channels, after trim DAC calibration, are shown in Figure 18. The shape of the pulse is similar to that obtained without the sensor (Figure 14) but the baseline is not completely flat. Residual effects of the 40 MHz disturbance mentioned previously is still visible, although the amplitude is very small, of the order of one LSB. This observation is in agreement with SALT simulations, which showed that it was not possible to completely remove this effect. Since the disturbance is synchronous with the system clock, it will be seen as a constant baseline and subtracted during signal processing in the DSP. Consequently, its effect on the signal amplitude measurement should be negligible. The pulse amplitude after sensor connection is about 24 LSB, similar as for the bare chip. It shows that for the foreseen sensor capacitance range the output amplitude does not change significantly. Signal-to-noise ratio for the setup with the sensor is above 20 and, as expected, is lower than for the bare chip.

### 3.4. Discussion

The development of low-power, large (≥32 channels), complex readout ASICs with on-chip fast ADC in each channel has only recently become possible. Examples of such ASICs include the 32-channel SAMPA [10] ASIC for gaseous detectors in the ALICE experiment at LHC or the 128-channel SMX2 ASIC for silicon and gaseous tracking detectors for the future CBM experiment at FAIR [41]. Other prototype ASICs, presented so far only at conferences, are the 64-channel VMM [11] for tracking detectors at the upgraded ATLAS experiment at the LHC, the 32-channel FLAME [9] for the luminosity calorimeter in the future linear collider, or the 72-channel HGCROC [12] chip being developed for a future high granularity calorimeter in the CMS experiment at LHC. Table 3 compares some of the main parameters of the SALT chip to those of SAMPA, and SMX2 ASICs.

The table shows the state-of-the-art for readout ASICs with on-chip fast ADCs in HEP experiments, and is not intended for direct comparison as each ASIC was designed according to different detector specifications and requirements. In the case of the SAMPA a higher ADC resolution was required, while for the SMX2, in addition to the amplitude, a time measurement was required. As can be seen from the table, the advantage of the SALT chip is the high sampling rate and fast shaping, allowing it to cope with 100% channel occupancy.

## 4. Conclusions

A 128-channel readout ASIC, SALT, was designed and fabricated in CMOS 130 nm process to readout silicon strips in the UT detector of the LHCb experiment. SALT is a SoC type ASIC, containing an analogue front-end and 40 MSps 6-bit ADC in each channel followed by a DSP, serial data transmission and many other blocks, including PLLs, DLLs, I^2^C, TFC, DACs, SLVS and monitoring circuitry. All together, they provide all required functionalities on-chip. The SALT development represents the first ASIC with ADC implemented in each channel achieving the readout of all channels with a high speed (40 MSps) and low-power, which allows for collecting all data at an LHC bunch crossing rate of 40 MHz. Combined with fast signal shaping, it allows us to cope with full channel occupancy.

The prototypes of SALT were tested as bare chips and together with silicon sensors, confirming the expected functionality and fulfilling the specifications. Recently, following these positive results, assembly of the UT detector with the final readout system has begun.

## Figures and Tables

**Table 1:** Specifications of the SALT Application Specific Integrated Circuit (ASIC).

Variable	Specification
Technology	CMOS 130 nm
Channels per ASIC	128
Power dissipation per channel	<6 mW
Radiation hardness	30 Mrad TID + TMR against SEE effects
Sensor input capacitance	1.6–12 pF
Signal to Noise ratio	>10 for MIP
Input signal polarity	Both, positive and negative
Dynamic range	Input charge up to ∼30,000 e${}^{-}$
Pulse shape and tail	$T_{peak}∼$25 ns, tail after 2 × *T*${}_{peak}$ ∼5% amplitude
ADC bits	6 bits (5 bits for each polarity)
ADC sampling rate	40 MHz
DSP functions	Pedestal and common mode subtraction, zero suppression
Output data interface	Five serial links @ 320 Mbit/s (SLVS standard)
Slow controls interface	I^2^C

**Table 2:** Data packet format. Both parts Header and Data are aligned to 12-bit word; parity bit ’*’ is calculated as the exclusive or of all other bits in the header; *k* = 0, 1, 2, …, 66.

	Header				Data	
Packet	BXID	Parity	Type + Length			Comment
	*4-bit*	*1-bit*	*1-bit*	*6-bit*	*12*·*k-bit*	
*BxVeto*	*bxid[3:0]*	*	1	010001	—	BxVeto in TFC
*HeaderOnly*	*bxid[3:0]*	*	1	010010	—	Header in TFC
*BusyEvent*	*bxid[3:0]*	*	1	010011	—	*nHits* > 63
*BufferFull*	*bxid[3:0]*	*	1	010100	—	no space in mem.
*BufferFullN*	*bxid[3:0]*	*	1	010101	—	no space in mem.
*NZS*	*bxid[3:0]*	*	1	000110	Values	NZS in TFC
*Normal*	*bxid[3:0]*	*	0	*nHits*	Hits	Normal event
*Sync*	*bxid[11:0]*				*pattern*	Synch in TFC

**Table 3:** State of the art readout ASICs for HEP experiments with on-chip per channel ADCs (SST—Silicon Strip Tracker, TPC—Time Projection Chamber, MCH—Muon Chamber).

Chip Name	SALT	SAMPA	SMX2
	(This Work)	[10]	[8]
Detector type	SST	TPC/MCH	SST/MCH
Technology node [nm]	130	130	180
No of channels	128	32	128
Sensor capacitance [pF]	1.6–12	18.5, 40–80	≤50
Signal polarity	both	both	both
Shaping	complex poles&zeros	CR–RC${}^{4}$	CR–RC
Peaking time [ns]	25	160, 300	80–270
Sampling rate [MHz]	40	5–10	0.5
ADC architecture	SAR	SAR	Flash
ADC resolution [bit]	6	10	5
Power/channel [mW]	3.5	8.3	8

## Data Availability

Data available on request. The data presented in this study are available on request from the corresponding author.
